# Preparation and characterization of carbon nanofluid by a plasma arc nanoparticles synthesis system

**DOI:** 10.1186/1556-276X-6-293

**Published:** 2011-04-05

**Authors:** Tun-Ping Teng, Ching-Min Cheng, Feng-Yi Pai

**Affiliations:** 1 Department of Industrial Education, National Taiwan Normal University, No. 162, Sec. 1, He-ping E. Rd., Da-an District, Taipei City 10610, Taiwan; 2 Department of Mechatronic Technology, National Taiwan Normal University, No. 162, Sec. 1, He-ping E. Rd., Da-an District, Taipei City 10610, Taiwan

## Abstract

Heat dissipation from electrical appliances is a significant issue with contemporary electrical devices. One factor in the improvement of heat dissipation is the heat transfer performance of the working fluid. In this study, we used plasma arc technology to produce a nanofluid of carbon nanoparticles dispersed in distilled water. In a one-step synthesis, carbon was simultaneously heated and vaporized in the chamber, the carbon vapor and particles were then carried to a collector, where cooling furnished the desired carbon/water nanofluid. The particle size and shape were determined using the light-scattering size analyzer, SEM, and TEM. Crystal morphology was examined by XRD. Finally, the characterization include thermal conductivity, viscosity, density and electric conductivity were evaluated by suitable instruments under different temperatures. The thermal conductivity of carbon/water nanofluid increased by about 25% at 50°C compared to distilled water. The experimental results demonstrated excellent thermal conductivity and feasibility for manufacturing of carbon/water nanofluids.

## Introduction

As industrial and technological products demand higher standards of function and capacity, the problem of heat dissipation from electrical appliances becomes a significant issue. To ameliorate this problem, there are four approaches commonly taken: (1) enlarge the heat exchanger area and structure, (2) fabricate the heat exchanger using materials with higher thermal conductivity, (3) increase the working fluid flow rate to the heat exchanger, and (4) improve the heat transfer performance of the heat exchange working fluid. Of these methods, enlargement of the heat exchanger area has reached a physical limit. Increasing the flow rate of heat exchange would create problems of volume, power consumption, and noise from the fan and pump. The thermal conductivity of copper and aluminum heat exchangers are quite high, and the addition of precious metal to improve thermal conductivity further would incur a tremendous increase in the heat exchanger cost. Therefore, we consider that in order to increase heat dissipation, the most feasible approach is to improve the heat transfer performance of the heat exchange working fluid.

The use of nanofluids to improve the heat-transfer performance of heat exchange working fluids deserves consideration. In 1995, Choi [1] became the first person to use the term "nanofluid" to describe a fluid containing nanoparticles. Nanofluid manufacture involves dispersing metallic and non-metallic nanomaterials with high thermal conductivity, into a suitable "working fluid" such as engine oil, water, ethylene glycol, *etc*., to enhance the heat transfer performance of traditional fluids [2]. According to literature reports, the thermal conductivity of a nanofluid is strongly dependent on the volume fraction and properties of the added nanoparticles [3,4]. In addition, for the addition of a given volume of particles, the solid-liquid surface contact area between nano-scale particles and the suspension fluid is greater than that for micro-scale particles. Hence, the size and shape of the particles added will have a significant effect on thermal conductivity and heat transfer characteristics [1,5-12].

Nanofluids preparation generally follows one of two methods: a one-step and a two-step synthesis. The so-called "one-step synthesis" produces nanofluids by synthesizing the nanoparticles directly into a suspending fluid, while the two-step process produces the nanoparticles and then disperses them in a bulk liquid to form a stable suspension, as separate processes.

Many variations on the one-step synthesis of nanofluids exist. Akoh *et al*. [13] used the VEROS method to prepare nanofluids in a one-step by applying vacuum evaporation to a running oil substrate. Wagener *et al*. [14] adopted magnetron sputtering to improve the VEROS technique, and succeeded in developing an effective preparation of Ag, Fe nanofluids. Zhu *et al*. [15] employed a new chemical method to prepare Cu-ethylene glycol nanofluids from reaction under microwave irradiation. Eastman *et al*. [16] also improved on the VEROS technique, by using low-temperature and low-pressure conditions, and letting Cu vapor directly contact and flow with low-vapor-pressure ethylene glycol fluid, causing the Cu vapor to condense directly in the fluid to form Cu nanofluid. Lo *et al*. [17] used a submerged arc nanoparticle-synthesis system to prepare Cu-based nanofluids. Lo *et al*. let Cu vapor, formed by electric arc discharge, directly condense in low-temperature and low-pressure deionized water, or ethylene glycol, to form CuO and Cu nanofluids. These researchers also used this method to produce Ni nanomagnetic fluids [18], and achieved good results. Chang *et al*. [19] synthesized an Al_2_O_3 _nanofluid, with high suspension stability, using a modified plasma arc system. The vaporized metallic gas mixed thoroughly with the pre-condensed, deionized water, to form an Al_2_O_3_/water nanofluid. The average particle size was in the range 25-75 nm. Hwang *et al*. [20] employed a modified magnetron sputtering system to produce Ag/silicon oil nanofluids. The Ag nanoparticles were relatively uniform with primary size less than 5 nm. Kumar *et al*. [21] fabricated copper nanofluids, of metallic copper dispersed in ethylene glycol, using sodium hypophosphite as reducing agent and conventional heating. Wei *et al*. [22] applied chemical solution methods to synthesize cuprous-oxide (Cu_2_O) nanoparticles in water, to form Cu_2_O nanofluids. Abareshi *et al*. [23] produced magnetite Fe_3_O_4 _nanoparticles by a co-precipitation method at various pH values. The concentration was around 0.25-3.0 vol.%. Generally, the one-step synthesis has the advantage that nanoparticles form directly in the bulk liquid. Normally, this method contains an intrinsic sorting mechanism, in which excessively large particles settle by static placement, and the supernatant, containing finer nano-sized particles as the dispersion, simply collected. This approach provides nanofluids with good suspension properties. Unless required by the preparation process, there is no need to add any dispersant or surfactant to improve the dispersion, and thus, not interference will arise from the addition of such additives. However, a disadvantage of the one-step method is that preparation conditions influence the size, shape and concentration of nanoparticles, the range of particle size distribution is broad, and an accurate control of the concentration is difficult.

Considering reports of two-step nanofluid formation, there are many accounts of Al_2_O_3 _nanofluid preparation using ultrasonic dispersion [16,24,25]. Murshed *et al*. [26] employed ultrasonic dispersion to prepare TiO_2_/water nanofluid, and applied the same method to prepare Au, Ag, SiC, and carbon nanotube nanofluid. In general, two-step syntheses are more suitable for the preparation of oxide nanofluids, but are less appropriate for the preparation of metallic nanofluids. Wen and Ding [27] used a high shear homogenizer to solve an agglomeration problem with TiO_2 _nanoparticles. Operating the homogenizer at 24,000 rpm, with a shear rate of 40,000 s^-1 ^disrupted nanoparticle agglomeration and provided an adequate dispersion of nanoparticles with narrow size distribution. Nevertheless, although this method improved on the agglomeration problem, it was still unavailable to acquire the particle size as observed by SEM and TEM. Choi *et al*. [28] used ZrO_2 _bead milling in a vertical, super-fine grinding mill, to mix Al_2_O_3 _and AlN with transformer oil at volume fractions up to 4%, and added *n*-hexane to regard as dispersant in order to keep good suspension. Hwang *et al*. [20] treated carbon black (CB)/water, and Ag/silicon oil nanofluids, to various two-step procedures, using stirrer, ultrasonic bath, ultrasonic disrupter and high-pressure homogenizer methods in order to achieve small particle size, with good dispersion. The high-pressure homogenizer produced average CB and Ag particle diameters of 45 and 35 nm, respectively. Moosavi *et al*. [29] demonstrated a two-step synthesis of ZnO nanoparticles, by mixing ethylene glycol and glycerol with the aid of a magnetic stirrer. Moosavi *et al*. added ammonium citrate to act as a dispersant, and enhance stability of the suspension. This method produced a mean ZnO particle size of 67.17 nm.

Generally, two-step methods are simpler than one-step methods, because the nanoparticles may either be self-made, or purchased, then added to a bulk liquid to form nanofluids. However, in the process of addition, agglomeration can occur easily, resulting in poor suspension, thus, two-step methods often require dispersion methods such as ultrasonic sonication, mechanical stirring, a homogenizer, or the addition of a surfactant or dispersant, to disrupt agglomeration and provide dispersion and stabilize the suspension. The advantages of two-step syntheses are facile and rapid preparation of large volume nanofluids, greater control over nanoparticle concentration and narrower particle size distribution is than that of single-step syntheses.

In this study, we employed a plasma arc system to produce a carbon/water nanofluid with stable suspension, in a one-step process, without addition of any dispersant or surfactant. We fully characterized the microstructure, particle size distribution, and fundamental properties by suitable instrumentation, in order to demonstrate the feasibility of the process described herein.

## Preparation of carbon/water nanofluid by plasma arc

**Figure 1:**
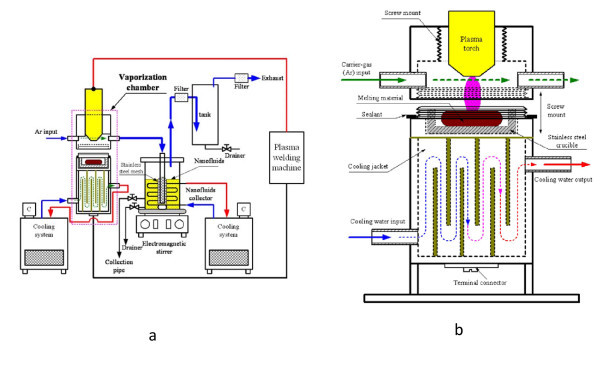
**Schematic diagram of the synthesis system for carbon/water nanofluid**.** (a)** The synthesis system for carbon/water nanofluid. **(b)** The vaporization chamber in the synthesis system.

The carbon/water nanofluid in this study was prepared by the plasma arc system [19], which belongs to one-step synthesis system. Figure 1 shows a schematic layout of the carbon/water nanofluid synthesis. Plasma arc welding equipment (400 GTS, Thermal Arc, Thermadyne, St. Louis, MO, USA) provided the heat source, and a vaporization chamber, cooling system, and collection system completed the system. The plasma arc provided the extreme high temperature inside the vaporization chamber, which melted and evaporated the graphite rods. Using this setup, we could control for working current, pulse frequency, and plasma gas and argon (Ar) carrier-gas flow rates. The pressure differential produced between the vaporization chamber and collection chamber induces vaporized carbon to move into the collection chamber. The nanofluid collection system and cooling system pre-cools distilled water to maintain a constant 3-5°C during the collection of nanofluid and to further suppress excess particle growth and clustering.

The low temperature of the working liquid (distilled water) instantly condenses the vaporized carbon to form nanoparticles, and the magnetic stirrer and stainless steel mesh thoroughly mix the resulting nanofluid, which will be induced out to form stable carbon/water nanofluid by collection pipe. Carbon nanoparticles suspended in cold distilled water have fewer interactions, so less aggregation occurs, resulting in smaller nanoparticles. Finally, we conducted an examination of the collected nanofluids material properties.

## Method and procedure for characteristic experiments

### Experimental procedure

All the completed experimental samples had to be statically placed for 48 h to confirm suspension performance, and to be identified concentration of carbon/water nanofluid changes less than 5% in a fixed depth of the container by using the spectrometer. For the particle size analysis, we used transmission electron microscope (FEI-TEM, Tecnai G^2 ^F20, Philips, Holland, the Netherlands) and a field emission scanning electron microscope (FE-SEM, 1530, LEO, Carl Zeiss Smt Ltd., Cambridge, UK) to identify microstructural properties. The suspended particle size and zeta potential of carbon/water nanofluids were measured using a light-scattering size/zeta potential analyzer (Zetasizer Nano ZS, Malvern Instruments, Worcestershire, UK) so as to determine clustering and suspension performance. Regarding the analysis of materials, the dry nanoparticles were obtained by centrifuge and heating the nanofluid to the appropriate speed and temperature. The crystalline phase was determined by X-ray Diffraction (XRD, APEX II, Kappa CCD, Monrovia, CA, USA). All peaks were measured by XRD and assigned by comparison with those of the joint committee on powder diffraction standards data (PCPDFWIN 2.4, JCPDS-ICDD, Newtown Square, PA, USA) [30]. Density, electric conductivity, viscosity, and thermal conductivities were measured by a density meter (DA-130N, KEM, Tokyo, Japan), rheology meter (DVIII+, BROOKFIELD, Middleboro, MA, USA), electric conductivity meter (CD-4306, Lutron Electronics Co., Inc., Taipei, Taiwan) respectively, and a thermal property analyzer (KD-2 Pro, Decagon Devices, Inc., Pullman, WA, USA) was used for determination of carbon/water nanofluids properties at various temperatures.

### Data analysis

The weight fraction (*ω*) of the carbon/water nanofluid is given by Eq. 1, with bulk fluid density (*ρ*_bf_), nanoparticle density (*ρ*_p_), and nanofluid density (*ρ*_nf_) [4,31]:(1)

The volume fraction (*ϕ*) of the carbon/water nanofluid is given by Eq. 2, with bulk fluid weight (*W*_bf_), nanoparticle weight (*W*_p_), and nanofluid weight (*W*_nf_):(2)

Equation 2 can be used to convert the weight fraction to volume fraction in order to compare the experimental results with the relevant literatures. However, it should be noted that the density is affected by temperature, so the volume fraction will be slightly changed by temperature.

For easy comparison of experimental data after changing the carbon/water nanofluid (*D*_nf_), all data obtained with the distilled water is used as baseline values (*D*_bf_); that is, experimental data obtained after the carbon/water nanofluid is used to compare with baseline values. The differences before and after changing the carbon/water nanofluid is presented as proportions (*R*), it can be calculated as follows:(3)

### Uncertainty analysis

In this study, the uncertainty of the experimental properties results was determine from the measurement deviation of the parameters, such as density, viscosity, electric conductivity, thermal conductivity, weight and temperature, as described by Kulkarni *et al*. [32]. In the density experiment, the density was determined from readings of the density meter (*ρ_t_*), resistance temperature detector (RTD, pt-100) of isothermal unit (*T*).(4)

The precision of the density meter was ±1%. The precision of the RTD was ±0.5°C. Hence, the uncertainty of the density experiment was less than ±2.7%.

In the viscosity experiment, the viscosity was determined from readings of the rheology meter (*μ_t_*), RTD (pt-100) of isothermal unit (*T*).(5)

The precision of the rheology meter was ±1%. The precision of the RTD was ±0.5°C. Hence, the uncertainty of the viscosity experiment was less than ±2.7%.

In the electric conductivity experiment, the electric conductivity was determined from readings of the rheology meter (*e_t_*), RTD (pt-100) of isothermal unit (*T*).(6)

The precision of the electric conductivity meter was ±3%. The precision of the RTD was ±0.5°C. Hence, the uncertainty of the electric conductivity experiment was less than ±3.9%.

In the thermal conductivity experiment, the thermal conductivity was determined from readings of the thermal property analyzer (*k_t_*), RTD (pt-100) of isothermal unit (*T*).(7)

The precision of the thermal property analyzer was ±5%. The precision of the RTD was ±0.5°C. Hence, the uncertainty of the thermal conductivity experiment was less than ±5.6%.

## Results and discussion

**Figure 2:**
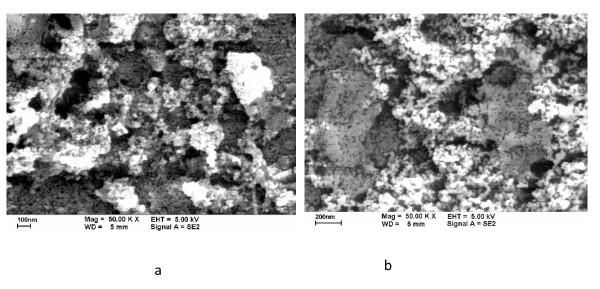
**SEM image of carbon nanoparticles**. **(a)** NC-70, **(b)** NC-80.

**Figure 3:**
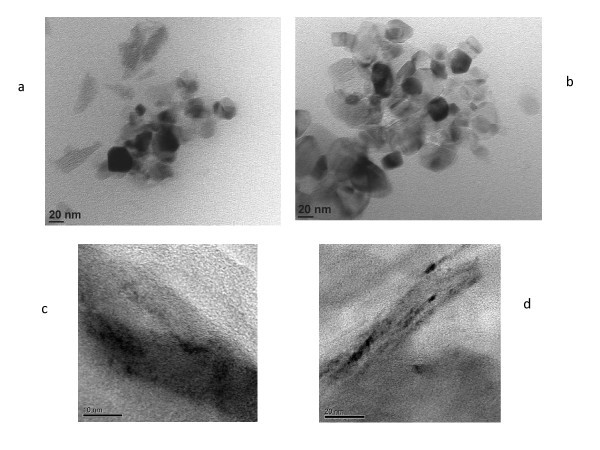
**TEM image of carbon nanoparticles**.** (a)** NC-70, **(b)** NC-80, **(c) **Edge of NC-70, **(d)** Edge of NC-80.

We maintained the working currents at 70 A (NC-70) and 80 A (NC-80). Table 1 lists the fabrication parameters and partial experimental and calculated results for the carbon/water nanofluid. Figures 2 and 3 are respectively the SEM and TEM photographs of carbon nanoparticles. From the figures, these can show that the nanoparticles are irregular in shape, and the nanoparticles occurred in an aggregate phenomenon. In addition, Figure 3c, d is the TEM photograph for the edge of carbon nanoparticles. The thickness of carbon nanoparticles is much smaller than its length and width in the photographs. Overall, the shape of these nanoparticles is of the shape of flakes (d-Spacing about 0.35 nm).

**Table 1:** List of fabrication parameters and properties for carbon/water nanofluid

Name	NC-70	NC-80
Working currents (A)	70	80
Working voltage (V)	24.3~24.7	26.2~26.8
Working power (kW)	1.70~1.73	2.10~2.15
Pulse frequency (Hz)	25	25
Plasma Ar (L/min)	1.5	
Shield Ar (L/min)	9	
Carrier-gas/Ar (L/min)	18	
Distilled water volume (ml)	500	
Manufacturing time (s)	1,000	
Particle size (Z-average, nm)^a^	244.4	284.6
Zeta potential (mV)^a^	-24.4	-21.2
Concentration (wt.%)^a^	0.02	0.04

^a^Data are measured and calculated at 25°C.

**Figure 4:**
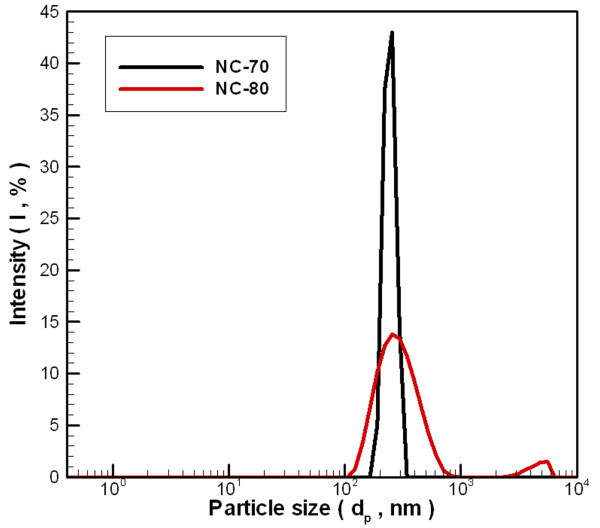
**Particle size distribution of carbon/water nanofluid**.

This study used the light-scattering size/zeta potential analyzer to determine the average nanoparticle size when suspended in distilled water. Figure 4 shows the particle size distribution for the carbon nanoparticles suspended in distilled water. Table 1 shows that for nanofluids at a working current of 70 A, the z-average particle size is 244.4 nm and the zeta potential is -24.4 mV. The distribution only has a single-peak, and dispersion is good. For nanofluids with a working current of 80 A, the z-average particle size is 284.6 nm, and a double-peak distribution appears at 298.9 and 4,590 nm. The zeta potential is -21 mV. From the distribution of measured values, we see that the secondary particle size is far greater than the primary particle size, as measured by SEM and TEM. This is mainly because agglomeration continues to occur to the suspended nanoparticles in distilled water and the tested particle size is greater than the particle size as observed by SEM and TEM.

**Figure 5:**
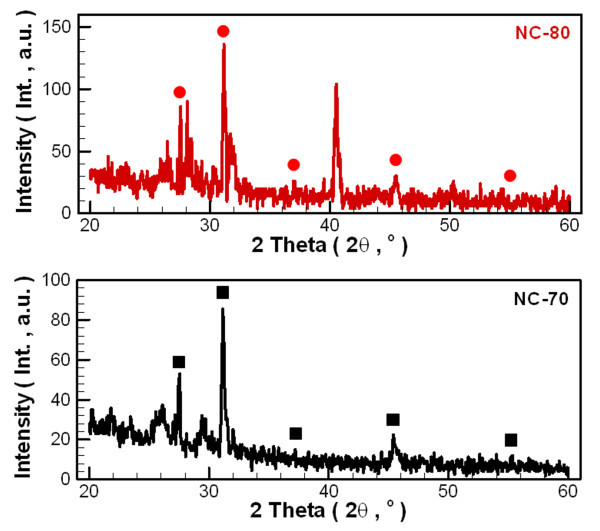
**X-ray diffraction pattern of carbon nanoparticles**.

**Figure 6:**
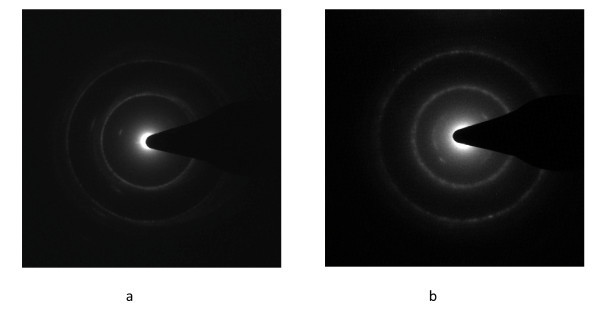
**TEM diffraction patterns of carbon nanoparticles**. (a) NC-70, (b) NC-80.

Figure 5 shows XRD patterns of the carbon nanoparticles obtained by centrifuging and heating of the nanofluids. We found that the major component of both the NC-70 and NC-80 fluids was carbon by comparing with PCPDFWIN data (PDF#460945) [30]. The diffraction peak intensity is not high, so the major structure of nanoparticles should belong to the multi-layer sheet of amorphous carbon. Therefore, changes in the process parameters did not significantly affect the materials' crystallization phase. Also, from the TEM diffraction patterns (Figure 6) of these carbon nanoparticles, non-crystalline structure can be seen.

**Figure 7:**
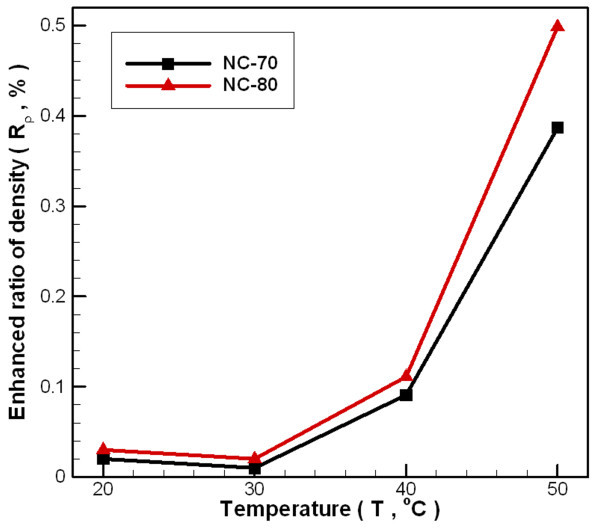
**Dependence relationship between temperatures and density enhanced ratio of carbon/water nanofluid under different fabrication parameters**.

Figure 7 shows changes in the density ratio of carbon/water nanofluids to that of distilled water at various temperatures. Between the enhanced ratio of density and the temperature difference, there is no obvious trend in the ratio due to heating, mainly because the nanofluid is a solid-liquid mixture. The thermal expansion rate of the bulk liquid is different from that of the nanoparticles, thus providing an inconsistent trend in density change. The density of carbon was measured by weighing after drying at fixed weight of nanofluid and calculated by Eq. 1, and the density of carbon nanoparticles was about 1,900 kg/m^3 ^to approximately 2,050 kg/m^3^. For a concentration of about 0.02 wt.% (NC-70) and a temperature in the range of 20-50°C, the density increases by 0.01-0.39%. For a concentration of about 0.04 wt.% (NC-80), the increase in density is 0.02-0.50%. The minimum increase in density ratios for both samples occurs at 30°C. The scope of the experimental deviation is limited because density change is not obvious.

**Figure 8:**
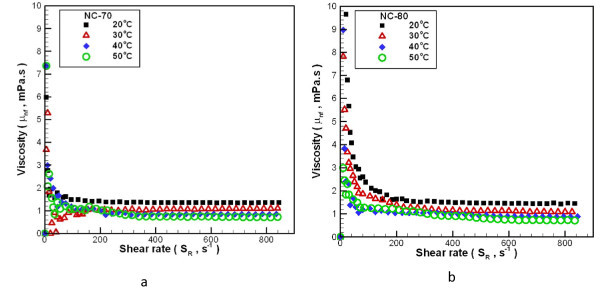
**Dependence relationship between shear rate and viscosity of carbon/water nanofluid under different temperatures**. (a) NC-70, (b) NC-80.

**Figure 9:**
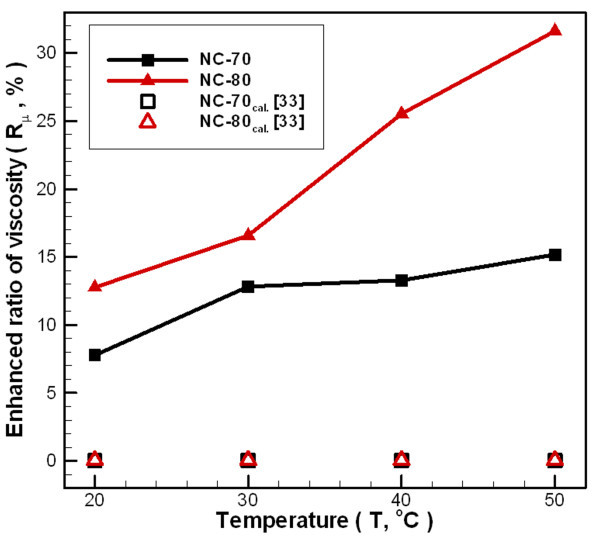
**Dependence relationship between temperatures and viscosity enhanced ratio of carbon/water nanofluid under different fabrication parameters**.

The viscosity of the carbon/water nanofluid as a function of shear rate, between 20°C and 50°C is shown in Figure 8. The viscosity of the carbon/water nanofluid is dependent on the shear rate over the entire measured temperature range. The addition of as little as 0.02 wt.% (NC-70) or 0.04 wt.% (NC-80) carbon nanoparticles to the distilled water results in carbon/water nanofluid displaying non-Newtonian behavior (shear thinning). Carbon/water nanofluids display Newtonian behavior with higher shear rate (*S*_R_>350 s^-1^), but the temperature of NC-80 is greater than 40°C. Additionally, the rheological properties of carbon/water nanofluid approach Newtonian behavior and increase carbon/water nanofluid concentrations at low temperatures. This trend occurs because viscosity reduces as water temperatures increase, so the added nanoparticles will increase the fluid internal shear stresses that results to the observed nanofluid viscosity. Adding more nanoparticles would produce a similar effect. Figure 9 shows the change in viscosity ratio for carbon/water nanofluids compared to distilled water at various temperatures and under different process parameters. In general, nanofluid viscosity increases with increasing nanoparticle loading in the bulk liquid. For an NC-70 concentration of 0.02 wt.% and within a temperature range of 20-50°C, the viscosity ratio increases by 7.77-15.17%. For an NC-80 concentration of 0.04 wt.%, the viscosity ratio increases by 15.76-31.63%. In addition, Figure 9 shows the calculated results of Einstein's model [33] (Eq. 8) in comparison with the experimental results that show a serious underestimation, which may be results from the material properties and aggregation of carbon nanoparticles [34]. From the above results, it can be found that the viscosity of carbon/water nanofluid is much higher than that of the water. When the carbon/water nanofluid was applied to heat exchange, pressure drop of pipeline and energy consumption of pump-related issues must be considered in particular in the future.(8)

**Figure 10:**
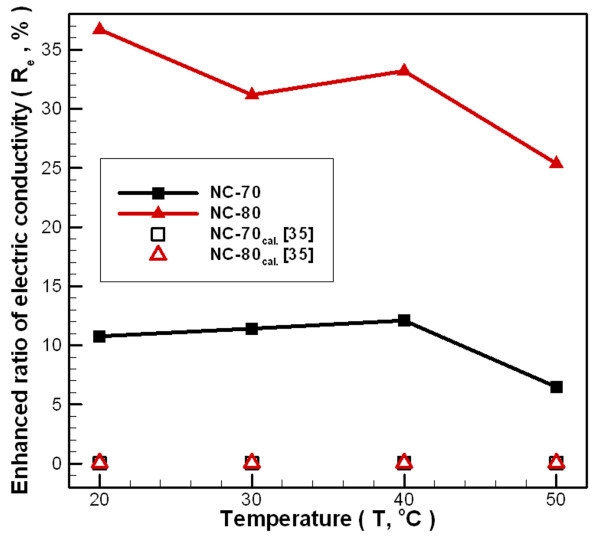
**Dependence relationship between temperatures and electric conductivity enhanced carbon/water nanofluid ratio under different fabrication parameters**.

Figure 10 shows the change in ratio of the nanofluid electric conductivity to distilled water at different temperatures. There is no dramatic change observed in electrical conductivity over the temperature test range of 30°C, since the temperature range is small. When the NC-70 concentration is 0.02 wt.% and the temperature of carbon/water nanofluid is in the range of 20-50°C, the change in electric conductivity ratio increases by 6.48-12.10%. For an NC-80 concentration of 0.04 wt.%, the change in electric conductivity ratio increases by 25.37-36.71%. The minimum enhanced ratios of electric conductivity for the two samples occur at 50°C. Comparing the experimental results with literature, this study used the model of Cruz *et al*. [35] modified from Maxwell's model [36] for analysis and comparison. Because the electric conductivity of carbon is much higher than that of the distilled water and that *α *is greater than one (*α *= (*e*_p _/*e*_bf_) ≫ 1), the principle of highly conducting particles (Eq. 9) is chosen to be compared with the experimental results of this study. Figure 10 shows a considerable underestimation while comparing calculation results with experimental data under most conditions. Because the Maxwell's model [36] is suitable only for fluids with large-size (micrometer or millimeter) particles dispersing [37-39], underestimation of the conductivity increases in nanofluid. Apart from the concentration and electric conductivity of particles and fluids, the effective electrical conductivity of nanofluids exhibits a complex dependence on the electrical double layer interactions [40,41], ionic concentrations, and other physicochemical properties which is not effectively captured by the Maxwell's model. Furthermore, this phenomenon of underestimation may result from the lower solid-liquid interface resistance due to high surface wetting of carbon nanoparticles by one-step synthesis, which results in the electric conductivity of carbon/water nanofluids with a higher enhancement.(9)

**Figure 11:**
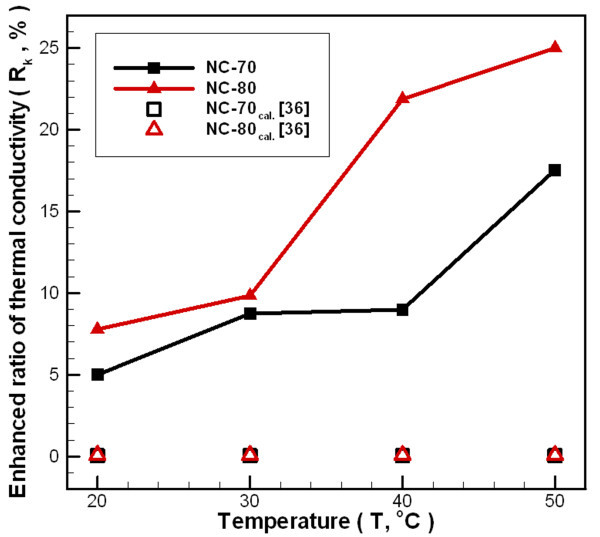
**Dependence relationship between temperatures and thermal conductivity enhanced carbon/water nanofluid ratio under different fabrication parameters**.

Figure 11 shows the change in thermal conductivity ratio for nanofluid compared to distilled water, over a temperature range of 20-50°C. The figure reveals that as the temperature increases, the effect of increasing nanoparticle concentration on the thermal conductivity ratio is greater than the applied temperature change. Increasing both concentration and temperature increases the frequency of particle liquid collisions producing a near quasi-convection phenomenon. Increasing random collision behavior helps to increase the thermal conductivity of carbon/water nanofluids, but there are some researchers who believe that the above-mentioned factors to increase the thermal conductivity were not significant [42,43]. For a concentration of 0.02 wt.% (NC-70) and a temperature in the range of 20-50°C, the ratio of thermal conductivity increases by 5.0-17.54%. For a concentration of 0.04 wt.% (NC-80), the ratio of thermal conductivity increases by 7.78-25.0% compared to distilled water.

In addition, Figure 11 shows an underestimation (Eq. 10) between the Maxwell's model [36] and the experimental results. This is because the Maxwell's model only considers the spherical and larger particles with the volume fraction of particles added, liquid and solid thermal conductivity on thermal conductivity of nanofluid, and cannot cover all factors. Since this study is made of non-spherical carbon nanoparticles, Maxwell's equation will show an undervalue. Moreover, this study found that low concentrations of added nanoparticles caused by the thermal conductivity increase should be from the interfacial thermal resistance and the aspect ratio of the dispersed particles [43-45]. Since the carbon/water nanofluids was manufactured by one-step synthesis in this study, non-spherical carbon nanoparticles were dispersed in the water and condensation occurred, so the interfacial thermal resistance should be relatively low due to high surface wetting of carbon nanoparticles which can effectively enhance the thermal conductivity of carbon/water nanofluid. Furthermore, the carbon nanoparticles made in this study are flake shaped, in which the thickness of the nanoparticle is much smaller than the length and width respectively, and thus adding such nanoparticle to the liquid can increase the thermal conductivity of nanofluids [26,46].(10)

## Conclusions

Using plasma arc in a one-step synthesis successfully produced a carbon/water nanofluid. The resulting nanofluid displayed good suspension performance, and the addition of dispersants was unnecessary. Characterization included thermal conductivity, viscosity, density, and electric conductivity measurements at various temperatures. The thermal conductivity of the carbon/water nanofluid is increased to about 25% at 50°C compared to distilled water. In addition, the manufacturing machine has the potential to produce the nanofluid with a variety of materials in the future. In the aspect of optimal manufacturing parameters for nanofluid, it is worth having a further in-depth study.

## Competing interests

The authors declare that they have no competing interests.

## Authors' contributions

TPT and CMC designed the experiment. CMC and FYP fabricated the sample. TPT and CMC carried out the measurements. TPT analyzed the measurements. TPT and CMC wrote the paper. All authors read and approved the final manuscript.
